# Response to Hypothetical Social Scenarios in Individuals with Traumatic Brain Injury Who Present Inappropriate Social Behavior: A Preliminary Report

**DOI:** 10.3390/bs3010072

**Published:** 2013-01-24

**Authors:** Jean Gagnon, Anne Henry, François-Pierre Decoste, Michel Ouellette, Pierre McDuff, Sacha Daelman

**Affiliations:** 1 Department of Psychology, University of Montreal, Montreal, H3C 3J7, Canada; E-Mails: annehenry@videotron.ca (A.H.); frank0996@gmail.com (F.-P.D.); pierre.mcduff@umontreal.ca (P.M.D); sacha.daelman@umontreal.ca (S.D.); 2 Centre for Interdisciplinary Research in Rehabilitation of Greater Montreal (CRIR) H2H 2N8, Canada; E-Mail: m.ouellette@umontreal.ca (M.O); 3 Department of Psychology, University of Sherbrooke, Sherbrooke, H2V 2S9, Canada; 4 Centre de recherche en neuropsychologie et cognition (CERNEC), Montreal, H3C 3J7, Canada

**Keywords:** inappropriate social behavior, traumatic brain injury, decision making, social interaction

## Abstract

**Background:** Very little research thus far has examined the decision making that underlies inappropriate social behavior (ISB) post-TBI (traumatic brain injury). **Objectives:** To verify the usefulness of a new instrument, the Social Responding Task, for investigating whether, in social decision making, individuals with TBI, who present inappropriate social behavior (ISB), have difficulty anticipating their own feelings of embarrassment and others’ angry reactions following an ISB. **Methods:** Seven subjects with TBI presenting with inappropriate social behavior (TBI-ISB), 10 presenting with appropriate social behavior (TBI-ASB), and 15 healthy controls were given 12 hypothetical scenarios three times, each time ending with a different behavioral response. Subjects were asked to gauge the likelihood of their displaying the behavior in that situation (part A) and of it being followed by an angry reaction from the other or by feelings of embarrassment in themselves (part B). **Results:** TBI-ISB subjects scored higher than TBI-ASB and healthy controls on a scale of likelihood of displaying an ISB. Results regarding expectations of angry reactions from others and feelings of embarrassment after an ISB were similar among groups. Negative correlations between endorsement of an inappropriate behavior and anticipation of negative emotional consequences were significant for both TBI-ASB and control subjects, but not for TBI-ISB subjects. **Conclusions:** Results suggest that the TBI-ISB participants were likely to endorse an ISB despite being able to anticipate a negative emotional response in themselves or others, suggesting that there were other explanations for their poor behavior. A self-reported likely response to hypothetical social scenarios can be a useful approach for studying the neurocognitive processes behind the poor choices of individuals with TBI-ISB, but the task needs further validation studies. A comprehensive discussion follows on the underlying mechanisms affecting social behaviors after a TBI.

## 1. Introduction

### 1.1. Somatic Marker Hypothesis and Decision-Making Processes

Several studies have described adults with damage to ventromedial frontal cortices who developed abnormal social conduct and inadequate decision making and planning that repeatedly led to negative consequences [1,2]. These individuals’ generally well-preserved intellectual abilities contrasted with abnormality in their processes of emotion and feeling, such as not experiencing embarrassment induced by specific social contexts [3]. These deficits led to the development of Damasio’s somatic marker hypothesis [4], which proposes that a deficit in emotion and feeling plays an important role in impaired decision making. The central feature of this theory is that emotion-related signals (somatic markers) stored in the ventromedial prefrontal cortex assist cognitive processes in implementing decisions. Over time, emotions and their corresponding bodily changes become associated with particular situations and their past outcomes. When making decisions at a later time, these signals and their evoked emotions are consciously or unconsciously associated with their past outcomes and bias decision making toward certain behaviors and away from others [4]. Disruption of the somatic marking processes leads to inappropriate social behavior (ISB) and failure to behave altruistically. This is explained by the fact that the immediate benefits of deviating from social norms or behaving self-servingly are not balanced by somatically marked future consequences associated with guilt or social rejection [5]. 

The most compelling empirical evidence for this hypothesis comes from the Iowa Gambling Task (IGT). The essential feature of the IGT is that it simulates real-life situations in the way it factors in uncertainty, reward and punishment [3]. Participants choose between decks of cards yielding high immediate gain but larger future losses (bad decks) and decks yielding lower immediate gain but smaller future losses (good decks). In contrast with normal subjects and control subjects with brain injuries, patients with bilateral lesions of the ventromedial prefrontal cortex do not increase the number of their selections from the good decks, but persist in selecting more cards from the bad decks [6,7]. These results suggest that the performance profiles of subjects with ventromedial prefrontal cortex damage reflect their real-life inability to make advantageous decisions when faced with complex and uncertain situations [8]. Recent studies have applied new experimental paradigms to economic games to show the role of the orbitofrontal cortex in mediating social emotions such as regret [9] and guilt [10]. Impairment of these social emotions would be one way in which ventromedial prefrontal cortex damage impairs social behaviors. It has also been suggested that social emotions may serve to bridge the gap between decision making, in general, and social decisions, in particular [10]. Both guilt and regret are distinguished from more basic emotions like sadness or anger, in that they derive from one’s own present situation in combination with either another person’s situation (guilt) or forgone alternative outcomes (regret). Consistent with the somatic marker hypothesis, anticipatory somatic states of arousal may help forecast the negative emotional consequences (such as guilt and regret) of transgressing social norms, thereby motivating individuals, in subsequent choices, to avoid actions that generate such negative somatic states. However, those studies were not designed to measure subjects’ emotional responses directly during performance on economic games. Indeed, the conclusion about the specific role of social emotion in decision making was inferred from behavioral performance on the task. Koenigs and Tranel [11] encouraged direct measurement of subjects’ emotional responses in order to document the role of social emotion in decision-making processes in a social context. 

### 1.2. Inappropriate Social Behavior and Decision Making After a Traumatic Brain Injury

Although the neuropathology of traumatic brain injury (TBI) is characterized by focal impact lesions superimposed on a background of diffuse axonal injury, it is well established that the ventromedial prefrontal and orbitofrontal cortices generally are vulnerable to the effects of TBI [12]. Permanent changes in emotional and social behavior are among the most common and debilitating consequences of TBI and may negatively impact social relationships [13,14,15]. Individuals with TBI are much more seriously handicapped by these changes than by their residual cognitive or physical disabilities [16,17]. In a cohort of persons with brain injuries referred to a community-based behavior management service, Kelly *et al.* [18] found that the most frequent challenging behavior categories were verbal aggression and inappropriate social behavior (ISB). Among the 79 subjects with TBI in the sample, 81% displayed ISBs ranging from awkward, to annoying, non-compliant, unlawful and even dangerous, in increasing order of seriousness. At the same time, patients with TBI also have difficulty making deliberate and advantageous decisions, as measured with gambling tasks [19,20,21]. Gambling task performance is insensitive to TBI severity and not limited to patients with large frontal lesions, but is related to executive functioning and working memory. Moreover, self-, other-, and examiner-rated complaints of real-life difficulties with affective regulation (e.g., lethargy, poor temper control, overexcitement) were significantly correlated with gambling performance [19]. 

There is a growing recognition of the need to develop alternative methods of measuring emotional and social behavior changes after a TBI [22] and of the importance of having relevant ecological instruments to better understand which aspects of cognition are critical for competency in everyday activities [23]. Using neuropsychological tests designed to assess recognition of emotional expressions and understanding of other people’s mental states and of social situations, Milders *et al.* [22] found that, compared to matched healthy control subjects, subjects with TBI were impaired at recognizing facial and vocal expressions of emotion and detecting social *faux pas*, which require empathic knowledge about what other people find upsetting or insulting [24]. In a case study, Blair and Cipolotti [25] reported on a patient, J.S., with an orbitofrontal cortex lesion, who presented with an aberrant level of aggression and a callous disregard for others. In a series of experimental investigations in which his performance was contrasted with that of a second patient who also had a dysexecutive syndrome but no socially aberrant behavior, results indicated that J.S. displayed severe difficulty in emotional expression recognition and an autonomic response to angry and disgusted expressions. Also, J.S. experienced difficulties in attributing the emotions of fear, anger and embarrassment to story protagonists, as well as in identifying violations of social behavior. According to the authors, J.S. may have suffered damage to a system that would be activated by another’s angry expressions and would extinguish behaviors that transgress social norms. This system may be activated by representations of past situations associated with other people’s angry responses, such as staring or glowering, that could precede a sense of embarrassment. This system would be critical in situations where there are no explicit social rules but where appropriateness is a function of associations with other people’s reactions to a behavior. They assessed these difficulties through social situation tasks that depicted behavior that was either normative or a violation of norms and asked subjects to say how appropriate the behavior was. This type of task is designed to assess the subject’s judgment of the appropriateness of a behavior in a given social situation. However, to investigate social decision-making processes, a task designed to assess the response selection process (e.g., what the subject would most likely do or say in the given situation) would be more appropriate [26]. 

### 1.3. Goal and Hypotheses

Despite the prevalence of ISB and difficulty in decision making after TBI, little research has directly examined the decision-making processes underlying this behavior. One question open to further investigation is whether individuals with TBI who display ISB fail to anticipate social emotions, such as embarrassment, and others’ negative emotional reactions, such as anger, in their social decision making. To respond to this question, we developed a performance task with ecological face validity to evaluate the self-reported likely response to hypothetical social scenarios by individuals with TBI who display ISB. In constructing the task, we sought to recreate ambiguous and conflictual conditions such as are found in real social contexts. 

Given that persons with TBI who display ISB represent an especially precarious population from the psychological and neuropsychological standpoints, and that recruiting such subjects is often a challenge for researchers, our goal in the present study was to gather preliminary data to verify the usefulness of our methodological approach for studying the neurocognitive bases of their social behaviors. The main goal of the present study was to compare the performance on our Social Responding Task of individuals with TBI who display ISB to that of individuals with TBI who do not display those behaviors. We expected that subjects with TBI and ISB would score higher on a scale of likelihood of displaying such behaviors in a variety of social situations than would subjects with TBI without ISB. We also expected that subjects with TBI and ISB would score lower on two scales of likelihood of anticipating a negative emotional consequence (*i.e*., another person’s anger, a sense of personal embarrassment) after displaying an ISB. Finally, we expected to observe a relationship between the likelihood of displaying an ISB and the likelihood of anticipating a negative emotional consequence after displaying an ISB. 

## 2. Experimental Section

### 2.1. Participants

Seventeen subjects with TBI participated in this study, in two groups: TBI with inappropriate social behaviors (TBI-ISB; N = 7) and TBI with appropriate social behaviors (TBI-ASB; N = 10). The inclusion criteria for both groups were: (a) subjects had experienced a mild, moderate or severe TBI, according to the information in their medical records; (b) the TBI occurred more than six months prior to the data collection, to ensure the cognitive and behavioral sequelae were stable; (c) the TBI occurred when the subject was over the age of 16 years; (d) the subject was under the age of 60 years at the time of the study; and (e) subjects demonstrated sufficient language and memory capacity to accomplish the tasks; (f) had undergone specialized rehabilitation services in recent years; and (g) lived in the community. We excluded subjects who: (a) presented a mental health disorder at the time of the study, according to information in their medical chart; or (b) had severe intellectual disabilities that would prevent their being able to give free and informed consent, such as might be indicated by the existence of a psychosis or mental retardation, or being under legal curatorship, according to their medical records. In addition to these criteria, subjects in the TBI-ISB group also had to: (a) present a severe behavior disorder including ISB, as demonstrated by a formal behavioral evaluation; and (b) be under supervision or specialized treatment for a severe behavior disorder. 

**Figure 1:**
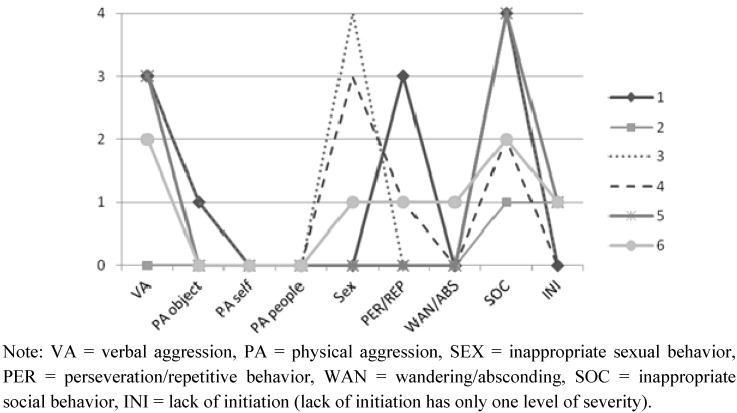
Maximum level of severity (from 0 to 4) for each TBI-ISB subject’s behavior on the Overt Behavior Scale (OBS).

Subjects in the TBI-ISB group were recruited through a specialized program for severe challenging behaviors at a rehabilitation center in Montreal and the provincial association for persons with brain injuries. Seven persons who met the inclusion criteria according to their treating professional or a member of their consulting team were referred to a member of the research team, who invited them to take part in the study. All agreed to participate. Subsequently, these persons were evaluated by their therapist using the Overt Behavior Scale (OBS) [27], an instrument designed to assess the types and severity of challenging behaviors, to ensure their behavioral profile matched the inclusion criteria (e.g., score of 1 or higher on the Inappropriate Social Behavioral scale); only one person was not tested, due to the unavailability of that person’s therapist. Figure 1 presents the behavioral profile for the subjects with TBI-ISB.

The subjects with TBI-ASB were recruited through the regular program for persons with TBI at the same rehabilitation center and another outside of Montreal. The program managers prepared lists of persons who had been treated for TBI in recent years, and letters introducing the study were sent to those persons. A member of the research team then telephoned them to present the project and invite them to participate. Those interested were then encountered to take part in the study. Among the 69 persons whose medical records were consulted, 58 met the selection criteria, and of the 44 who were successfully contacted by telephone and invited to participate, 10 agreed. In the end, for both TBI samples, a total of 17 French-speaking subjects met all requirements of the study (17/51 = 34%). Because the 10 TBI-ASB subjects were no longer receiving rehabilitation services, it was not possible to gather information from a therapist to complete the OBS for them. Consequently, OBS data are available only for the TBI-ISB subjects. 

Severity of the injury, established by the attending physiatrist and based both on the Glasgow Coma Scale score and on a positive cerebral scan, was matched between TBI groups. All subjects had received a diagnosis of either severe (TBI-ASB = 7; TBI-ISB = 5), moderate (TBI-ASB = 2; TBI-ISB = 1) or mild (TBI-ASB = 1; TBI-ISB = 1) TBI. Information on duration of coma and existence of post-traumatic amnesia was unavailable in the majority of cases. All subjects lived at home, except for three who lived in a foster home. 

In the TBI-ISB sample, the average age at the time of the TBI was 33.83 years (SD = 10.38) and the average post-injury elapsed time was 95.83 months (SD = 54.70). In the TBI-ASB sample, the average age at the time of the TBI was 36.00 years (SD = 15.27) and the average post-injury elapsed time was 59.70 months (SD = 51.84). The TBI-ISB sample consisted of seven men, whereas the TBI-ABS sample consisted of seven men and three women. 

For both TBI groups, information regarding sites of injury was obtained from written interpretations of computerized tomography scans found in the medical records. Table 1 presents clinical descriptions of both the subjects with TBI-ISB and those with TBI-ASB. Pre-TBI histories are very similar in both TBI samples. In the TBI-ISB sample, two subjects (29%) reported a pre-TBI history of either substance abuse or substance dependence and one subject (14%) reported a pre-TBI history of at least one Axis I psychiatric problem with or without a formal diagnosis (depression and anxiety disorder). In the TBI-ASB sample, two subjects (20%) reported a pre-TBI history of either substance abuse or substance dependence, and one subject (10%) reported a pre-TBI history of one Axis I psychiatric problem with or without a formal diagnosis (depression). No subjects in either group reported having a pre-TBI psychotic disorder.

**Table 1:** Clinical description of the TBI subjects.

Subjects	Group	CT scan or MRI results	Severity	Time since TBI (months)
1	TBI-ASB	DAI, B hemorrhagic contusions, B T focal hemorrhagic contusions	severe	57
2	TBI-ASB	Discopathy C5-C6. No clear signs of cerebral contusions	mild	12
3	TBI-ASB	L F subarachnoid hemorrhage, L F contusion, cerebral edema, B F hygromas, DAI located in R midbrain	moderate	33
4	TBI-ASB	R F epidural hematoma, T subdural hematoma	severe	93
5	TBI-ASB	L insular and thalamic contusions with cerebral edema and discrete mass effect, basal ganglia hematoma, DAI	severe	83
6	TBI-ASB	R T epidural hematoma, L subarachnoid hemorrhage, R P subdural hemorrhage, R P contusion	moderate	33
7	TBI-ASB	R T subdural hematoma, R thalamic hematoma, L T hemorrhagic contusion, R F encephalomalacia, cerebellar atrophy	severe	5
8	TBI-ASB	L F subdural hematoma with mass effect, L T intracerebral hematoma	severe	180
9	TBI-ASB	F R epidural and subdural hematoma, B F hemorrhage, F P hemorrhage	severe	75
10	TBI-ASB	DAI, cerebral anoxia	severe	26
11	TBI-ISB	B F, L T P and interhemispheric subdural hemorrhages, subarachnoid and intraventricular hemorrhages, DAI.	severe	101
12	TBI-ISB	R F contusion with mass effect, F cerebral edema, intracerebral and subdural hematomas, R F T and L F fractures of the skull	severe	22
13	TBI-ISB	Data unavailable	mild	na
14	TBI-ISB	R cerebral edema with cerebral herniation, F hemorrhage, R depressed fracture of the skull associated with intracerebral and subarachnoid hemorrhage	moderate	68
15	TBI-ISB	Cerebral oedema with F hemorrhagic contusion, R F and B T contusions	severe	155
16	TBI-ISB	R F P T subdural hematoma, B F T subarachnoid hemorrhage, cerebral edema, multiple facial fractures	severe	67
17	TBI-ISB	F and non-F contusions	severe	162

Note: DAI = diffuse axonal injury; L = left; R = right; B = bilateral; F = frontal, T = temporal; P = parietal.

Fifteen healthy control subjects took part in the study, matched to the TBI subjects by age. No control subject had a history of neurological illness or mental health disorder, based on a self-report questionnaire. Control subjects were recruited from the community and encountered by a member of the team in order to take part in the study. The control sample consisted of seven men and eight women. Average education was 14.2 years (SD = 2.49). An ANOVA analysis indicated that the ages of the subjects with TBI-ISB (M = 42.57; SD = 7.57), those with TBI-ASB (M = 41.30; SD = 14.28) and the healthy control subjects (M = 45.73; SD = 12.22) did not differ significantly (F(2,29) = 0.436, n.s.). Due to the unequal number of subjects per group, groups were not balanced in terms of gender. Informed consent was obtained from each participant in the study.

### 2.2. Materials

#### 2.2.1. Social Responding Task

This task, which we developed, consisted of 12 original scenarios presented in short written texts of about four or five lines each, illustrating social situations in which subjects were asked to identify with one of the characters (see Supplementary Material: 12 scenarios from the Social Responding Task). The scenarios involved a variety of social situations from everyday life that presented, to different extents, a conflict between satisfying one’s own needs or those of others, with the potential consequences of the action being more or less explicit. Scenarios were developed that would lead to expectations of angry responses in others and feelings of personal embarrassment after the display of an ISB. The task was developed based on six criteria: (1) the social situations had to be plausible in everyday life; (2) scenarios should not refer to social situations with very familiar social scripts that might provide indications on the usual, conventional way of behaving; (3) scenarios had to present a social interaction involving at least two persons; (4) they had to be written so that the subject could identify with the character speaking; (5) each scenario had to refer to a different social situation; and (6) the scenarios had to be comparable in the number of words and the number of persons presented. Two neuropsychologists with more than 10 years of experience working in a rehabilitation center with individuals with TBI were asked to read the scenarios to ensure they respected the six criteria; revisions were made based on their comments. 

The task consisted of two parts. In Part A, subjects were asked to read the scenario, which was followed by a behavior that was either socially appropriate or inappropriate, and then to indicate the extent to which they would have reacted in the same way if they had been in that situation. The inappropriate social behaviors proposed in the behavioral responses were inspired by those in the OBS scale [27]. Once constructed, the task underwent content validation for the behavioral responses with 14 healthy participants living in the community. For each scenario in the task, they were asked to assess the level of social adjustment represented by the behaviors illustrated on a scale from 1 (“very inappropriate”) to 4 (“very appropriate”). The results showed that the inappropriate social behaviors (M = 1.74/4; SD = 0.22) were judged as being significantly more inappropriate than the appropriate social behaviors (M = 2.27/4; SD = 0.24; *t*(13) = 9.277, *p* < 0.001). 

To simulate a decision-making task that would include both appropriate and inappropriate social behaviors, each scenario was presented three times, each time associated with a different behavior—two that were socially inappropriate and one, socially appropriate—for a total of 36 scenarios presented in a controlled random order (no more than two inappropriate behaviors consecutively). Associating each scenario with only one behavior at a time helped prevent any contamination effect of one response choice (more appropriate) on another (less appropriate), as sometimes happens in a multiple choice task. Subjects responded on a Likert scale ranging from 0 to 3 (0 = not at all likely; 1 = unlikely; 2 = likely; 3 = very likely). A response of “likely” or “very likely” was considered to indicate that the subject would have chosen this behavior in the given situation. 

In Part B, the same 12 scenarios were presented again, but this time only once. Each scenario was associated with a pre-selected behavior from Part A. Nine scenarios included an inappropriate social behavior, and three, an appropriate social behavior. Because one of our hypotheses concerned TBI-ISB subjects’ difficulties with anticipating negative consequences after an ISB, we included more inappropriate behaviors than appropriate ones to maximize the observations. In part B, the subjects were not asked to indicate the extent to which they would have reacted in the same way if they had been in that situation. Instead, after the presentation of the scenario associated with one behavior, two different emotional consequences were presented one after the other: an angry response from the other person, and feelings of personal embarrassment. Subjects were asked to indicate the extent to which they considered it likely that the behavior would be followed by each of the consequences. For their responses, they used the same Likert scale as in Part A. We decided to present the two scales for emotional consequences separately from the scale for behaviors (inappropriate and appropriate), that is, to separate Parts A and B of the task, so that the presentation of emotional consequences (angry reaction from others and feelings of personal embarrassment—Part B) would not influence the decision regarding the likelihood of displaying the behavior (Part A).

All the control subjects completed the task. In the TBI-ISB groups, all the subjects responded to all the scenarios in Part A, one subject responded to 67% of the scenarios in Part B, and another did not do Part B. In the TBI-ASB group, nine subjects responded to all the scenarios in Part A, one subject responded to 92% of the scenarios in Part A, two subjects responded to between 80% and 88% of the scenarios in Part B, and one subject did not do Part B. The main reasons for the subjects’ inability to complete the task were fatigue and impatience. 

Prior to the formal testing, all subjects with TBI were briefly assessed on certain cognitive and behavioral aspects that could influence their decision-making process. Because of the length of the performance task and the distinctive behavior profiles of the TBI-ISB subjects, a more comprehensive evaluation was judged to be unnecessary for the purposes of the present study. The following tests and questionnaires were used.

#### 2.2.2. Picture Completion Subtest from the Wechsler Adult Intelligence Scale-III (WAIS-III-PC)

The Picture Completion subtest from the WAIS-III [28] is one of the most widely used tests in neuropsychology [29]. This subtest is used particularly to assess visual attention; it consists of a series of 25 color images representing scenes or objects from everyday life, each of which is missing an important component. The subject is asked to identify the missing part that is most important. One point is given for each correct response, for a maximum of 25 points. All the subjects with TBI completed the task.

#### 2.2.3. Similarities subtest from the WAIS-III (WAIS-III-S)

This subtest from the WAIS-III is used to assess abstract verbal reasoning. It consists of 19 pairs of words (objects, foods, animals, concepts, *etc.*); subjects are asked to explain the similarities between the two items in each pair. Aside from the first five pairs, which are worth only one point, all other pairs are scored from 0 to 2 points depending on the quality of the subject’s response, for a maximum of 33 points. Two subjects in the TBI-ISB group and one in the TBI-ASB group did not do this task. 

#### 2.2.4. UPPS Impulsive Behavior Scale—Short version

The UPPS questionnaire, short version [30] measures four dimensions of impulsivity traits: Urgency (U), Lack of premeditation (Lpre), Lack of perseverance (Lper) and Sensation Seeking (SS). This scale is specifically designed to assess impulsivity changes after TBI and consists of 16 items rated on a 4-point Likert scale (1 = almost never, to 4 = almost always), which were adapted and transformed to assess impulsive behaviors at both the pre-morbid and current levels. The four subscales were created by selecting the four items from each dimension that had the highest item-total correlations in the original 45-item scale [31]. All the subjects with TBI completed this questionnaire. 

#### 2.2.5. Marlowe–Crowne Social Desirability Scale—Short form (MCSD)

The present study used the short version of the MCSD [32] to assess subjects’ tendency to respond to the Social Responding Task in a socially desirable manner. This test consists of 13 true–false items. One point is given for each desirable response, to a maximum of 13 points. Two subjects in the TBI-ASB group did not do the questionnaire.

### 2.3. Procedure

Subjects were met individually either at their rehabilitation center, at their long-term care residence, or at the offices of the provincial association. All the tasks and questionnaires were administered to all the subjects with TBI in the following order: Picture Completion, UPPS questionnaire, Social Responding Task Part A, Similarity, Social Responding Task Part B, Marlowe–Crowne Social Desirability Scale. For the healthy control subjects, the same order was used, except for Picture Completion and Similarity, which were not administered. Subjects with TBI were met once or twice, depending on their fatigue. 

## 3. Results

### 3.1. Analysis of the Social Responding Task

To allow direct links to be made between Parts A and B of the Social Responding Task, we retained for analysis only the results of the 12 scenarios in Part A that included the same behaviors as those in Part B (see Supplementary Material: Social Responding Task). The responses for inappropriate behaviors were separated from those for appropriate behaviors. A total score for each participant was obtained by calculating the mean of the scores on the four scales: (1) likelihood of displaying an inappropriate behavior; (2) likelihood of displaying an appropriate behavior; (3) likelihood that the other would react angrily; and (4) likelihood of feeling embarrassed. Given the small samples of the present study and the fact that the results on the “inappropriate behaviors” and “appropriate behaviors” scales were not normally distributed, Kruskal–Wallis tests were used to verify the overall group effect on the total score for each of the four scales (inappropriate behaviors, appropriate behaviors, angry reaction, and feelings of embarrassment) and Mann–Whitney U tests were used to compare specific pairs of groups. 

Also, given that each social situation presented in the scenarios had its own contextual and socio-affective elements that could have exerted different types of influence on behavioral decisions, we calculated the percentage of participants in each group who responded according to the four anchor points of the scale (0 = not at all likely; 1 = unlikely; 2 = likely; 3 = very likely) for each scenario, in order to compare the groups on every social situation and generate more specific hypotheses about the pattern of performance. Considering that many cells did not meet the minimum cell frequency for chi-square tests, these percentages were submitted to Cramer’s V test in order to establish a relationship between the two variables. The criteria for judging the effect sizes were those recommended for large tables: small = 0.07; medium = 0.21; large = 0.35 [33]. Adjusted standardized residuals were also calculated to identify the proportions that were significantly greater or lower than the expected cell frequency. 

To verify whether the choice of behavior could be explained by the anticipation of emotional consequences, the relationship between the likelihood of displaying inappropriate behaviors and the likelihood of experiencing an angry reaction from the other and/or personal embarrassment after displaying an ISB was submitted to two analyses. First, to establish a direct connection between a poor behavior choice and an inability to anticipate a negative response from others, and/or an inability to anticipate personal embarrassment, we calculated the percentage of participants in each group who endorsed the inappropriate behaviors in Part A and either: (1) also failed to anticipate an angry reaction, and/or (2) also failed to anticipate feelings of personal embarrassment following the same behaviors in Part B. This percentage was compared to the percentage of participants who did not endorse inappropriate behaviors and also failed to anticipate an angry reaction and/or feelings of personal embarrassment. Second, for each group separately, we used Spearman’s rank order correlation to describe the strength and direction of the relationship between the total score on the scale of likelihood of displaying inappropriate behaviors and the total score on the two scales of likelihood of experiencing a negative emotional consequence after displaying an ISB. 

As a control measure, we calculated for each TBI group the number of times a participant indicated “very likely” consecutively on the likelihood of displaying the behavior across the 36 scenarios in part A, in order to estimate the influence on the performance of a possible perseveration. We used the Kruskal–Wallis test to compare the groups on the longest sequence for each subject. Finally, to verify possible cognitive contributions to social decisions and anticipation of consequences, Spearman’s rank order correlations were performed between inappropriate behaviors, anticipation of angry reactions/feelings of embarrassment, and control measures. 

### 3.2. Results of the Social Responding Task

#### 3.2.1. Endorsement of Behaviors and Anticipation of Emotional Negative Consequences

Table 2 presents mean scores and Kruskal–Wallis test results on the four scales of the Social Responding Task. A statistically significant difference was found in the endorsement of inappropriate behaviors in Part A of the task across the three groups (χ^2^ (2, n = 32) = 8.78, p = 0.012), revealing that the TBI-ISB group scored higher than the TBI-ASB group (U = 11.50, z = −2.30, p = 0.21, r = 41) and the control subjects (U = 14.50, z = −2.69, p = 0.007, r = 48). No significant difference across groups was found on the other scales. 

**Table 2:** Mean scores (standard deviation) by group for inappropriate behavior, appropriate behavior, expectation of angry reaction from others and of feelings of embarrassment, and results of Kruskal–Wallis tests.

	TBI-ISB	TBI-ASB	Controls	Kruskal-Wallis (df = 2)	*p*
Inappropriate behaviors	1.43 (0.74)	0.61 (0.42)	0.44 (0.32)	8.78	0.012 *
Appropriate behaviors	2.29 (0.45)	2.73 (0.41)	2.64 (0.43)	4.55	0.103
Angry reaction	2.29 (0.45)	2.38 (0.19)	2.24 (0.56)	0.085	0.958
Embarrassment	1.83 (0.36)	2.23 (0.48)	2.20 (0.68)	3.75	0.153

Note: * *p* < 0.05.

Table 3 presents the percentage of participants by group who endorsed the inappropriate behaviors and the appropriate behaviors as “likely” or “very likely” across the scenarios and Cramer’s V test results. A large effect size of the correlation coefficient between the scale and the groups was found for seven out of nine scenarios associated with an inappropriate behavior, whereas the effect size was medium or small for the three scenarios with an appropriate behavior. The large effect sizes for the scenarios with inappropriate behavior were all significantly explained by a proportion of participants in the TBI-ISB group that exceeded the expected frequency, except for one scenario (scenario 3) where both TBI groups’ proportions exceeded the expected frequency. 

Regarding the percentage of participants by group who anticipated an angry reaction from the other person as being “likely” or “very likely” across the scenarios (results not included in Table 3), a large effect size of the correlation coefficient between the scale and the groups was found for three out of nine scenarios (scenarios 5, 6, 9) associated with an inappropriate behavior, and two out of three scenarios (scenarios 10 and 12) with an appropriate behavior. Only the large effect sizes for the scenarios with an appropriate behavior were significantly explained by a proportion of participants in the TBI-ISB group that exceeded the expected frequency. 

Finally, as for the percentage of participants by group who anticipated feelings of embarrassment as being “likely” or “very likely” across the scenarios (results not included in Table 3), a large effect size for the correlation coefficient between the scale and the groups was found for five out of nine scenarios (scenarios 2, 3, 4, 5, 8) associated with an inappropriate behavior, and for all of the three scenarios (scenarios 10, 11, 12) with an appropriate behavior. The large effect sizes for three scenarios (scenarios 2, 5, 8) with an inappropriate behavior were significantly explained by a proportion of participants in the TBI-ISB group that was lower than the expected frequency, whereas the large effect sizes for two scenarios (scenarios 11 and 12) with an appropriate behavior were significantly explained by a proportion of participants in the TBI-ISB group that was greater than the expected frequency. 

**Table 3:** Percentage of participants by group who endorsed inappropriate behaviors and appropriate behaviors as “likely” or “very likely” that they would have reacted in the same way had they been in that situation.

		TBI-ISB		TBI-ASB		Controls		
	Scenario	Likely	Very likely	Likely	Very likely	Likely	Very likely	Cramer’s V
Inappropriate behavior	1 (A16,B8)^1^	57.1^a^	14.3^ a^	10	0	0^ a^	0	0.55
	2 (A34,B2)	28.6^ a^	28.6^ a^	0	10	0	0	0.47
	3 (A5,B9)	57.1^ a^	0	22.2	22.2^ a^	0^ a^	0	0.53
	4 (A19,B1)	57.1	14.3^ a^	40	0	20	0	0.37
	5 (A26,B4)	42.9^ a^	28.6^ a^	10	0	6.7	0	0.50
	6 (A30,B7)	57.1^ a^	14.3	10	10	0^ a^	6.7	0.46
	7 (A33,B10)	28.6	0	20	0	6.7	0	0.28
	8 (A23,B5)	28.6^ a^	14.3^ a^	10	0	0	0	0.41
	9 (A12,B6)	0	14.3	0	10	0	0	0.28
Appropriate behavior	10 (A32,B12)	42.9	42.9^ a^	10	90	26.7	73.3	0.32
	11 (A3,B11)	28.6	42.9	20	70	26.7	60	0.26
	12 (A25,B3)	42.9	57.1	30	70	26.7	73.3	0.14

Note: ^a ^= adjusted standardized residuals with *p* < 0.05; Cramer’s V effect size: .07 = small; .21 = medium; 0.35 = large; ^1^ A=Part A; B=Part B; number=position of the scenario in Part A or Part B.

#### 3.2.2. Relationship Between Behaviors and Negative Emotional Consequences

Table 4 presents the percentage of participants by group who endorsed the inappropriate behavior in Part A and also failed to anticipate an angry reaction and/or feelings of personal embarrassment for the same inappropriate behavior in Part B, compared to the percentage who did not endorse the inappropriate behavior (in Part A) and failed to anticipate an angry reaction and/or feelings of personal embarrassment (in Part B). To demonstrate a link between a poor behavior choice and the inability to anticipate a negative response, the results must indicate that when participants endorsed an inappropriate behavior, they also failed to anticipate an angry reaction and/or feelings of personal embarrassment for the same behavior, as opposed to when they did not endorse the inappropriate behavior. This pattern of performance was found for each group. Indeed, the percentage of participants who endorsed the inappropriate behavior (Part A) and also failed to anticipate a negative emotional consequence following the same inappropriate behavior (Part B) was higher (10.3% to 66.7%) than the percentage of participants who did not endorse an inappropriate behavior but still failed to anticipate a negative emotional consequence (0% to 20%). In other words, among the participants who did not endorse the inappropriate social behavior in Part A, just a few of them still failed to anticipate a negative emotional consequence in Part B. However, two additional patterns should be pointed out. First, the proportion of participants who failed to endorse a negative consequence after an inappropriate behavior was higher for feelings of personal embarrassment than for an angry reaction. Second, the percentage of participants who endorsed an inappropriate behavior was much higher in the TBI-ISB group than in the TBI-ABS group and the control group (55%, 15% and 4% respectively). The fact that only 10.3% and 37.9% of the TBI-ISB subjects failed to anticipate an angry reaction and/or feelings of personal embarrassment, respectively, following a behavior that they endorsed more than half of the time (55%) suggests there are other explanations behind their poor choice of behavior. 

**Table 4:** Percentage (%) of participants by group who endorsed an inappropriate behavior (in Part A) and also failed to anticipate an angry reaction and/or feelings of personal embarrassment following the same behavior (in Part B), compared to the percentage of participants who did not endorse the inappropriate behavior and also failed to anticipate an angry reaction and/or feelings of embarrassment.

	Failed to endorse angry reaction			Failed to endorse feelings of embarrassment		
	TBI-ISB	TBI-ASB	Controls	TBI-ISB	TBI-ASB	Controls
Endorsed inappropriate behavior	3/29 (10.3%)	2/10 (20%)	2/6 (33.3%)	11/29 (37.9%)	6/11 (54.5%)	4/6 (66.7%)
Did not endorse inappropriate behavior	0/24 (0%)	7/56 (12.5%)	17/129 (13.2%)	5/24 (20%)	9/58 (15.5%)	23/129 (17.8%)
% of participants who endorsed an inappropriate behavior	29/53 (55%)	10/66 (15%)	6/135 (4%)			

Table 5 presents the Spearman’s rank order correlations by group between inappropriate and appropriate behaviors, expectations of angry reactions from others and feelings of personal embarrassment. There is a negative and significant correlation between the score on the inappropriate behavior scale and the score on the angry reaction expectation scale for the TBI-ASB group, and a negative and significant correlation between the score on the inappropriate behavior scale and the anticipation of personal embarrassment for the control subjects. No significant correlation was found for the TBI-ISB group, nor for the appropriate behavior scale.

**Table 5:** Spearman’s rank order correlations by group between inappropriate and appropriate behaviors, expectations of angry reactions from others and of feelings of embarrassment.

		Inappropriate behavior	Appropriate behavior
TBI-ISB	Angry reaction	−0.334	0.462
	Embarrassment	−0.493	0.250
TBI-ASB	Angry reaction	−0.859 **	0.507
	Embarrassment	−0.205	−0.449
Controls	Angry reaction	−0.142	0.138
	Embarrassment	−0.579 *	0.348

Note: * < 0.05; ** < 0.01.

### 3.3. Control Measures

Regarding estimation of the presence of perseveration on the same answer across the 36 scenarios during the performance of the TBI-ISB subjects, there was no statistically significant difference in the longest sequence of endorsement of the behavior as “very likely” between the two TBI groups’ participants (χ^2^ (1, n = 17) = 0.658, *p* = 0.417). 

The average scores on the other control measures for the TBI-ISB and TBI-ASB groups are presented in Table 6. A square root transformation was performed on UPPS Lack of perseverance to help normalize the distribution. No significant difference was observed between the two groups on all measures except WAIS-III-PC.

**Table 6:** Descriptive statistics on control measures for TBI groups.

	TBI-ISB	TBI-ASB	t tests	*p*
WAIS-III-PC	11.57/25 ( 5.71)	20.30/25 (3.62)	t(15) = 3.872	< 0.01
WAIS-III-S	13.80/33 (8.11)	22.33/33 (7.31)	t(12) = 2.016	n.s.
UPPS-U	1.75/4 (0.74)	1.95/4 (0.72)	t(15) = 0.561	n.s.
UPPS-Lprem	1.71/4 (0.64)	2.16/4 (0.66)	t(15) = 1.389	n.s.
UPPS-Lper	1.54/4 (0.53)	1.68/4 (0.94)	t(15) = 0.210	n.s.
UPPS-SS	2.57/4 (1.04)	2.20/4 (0.94)	t(15) = -0.768	n.s.
MCSD	6.33/13 (2.34)	8.88/13 (3.72)	t(12) = 1.463	n.s

Note: Mean on total score and standard deviation in parentheses.

Spearman’s rank order correlations between inappropriate behaviors, anticipation of angry reactions/feelings of embarrassment, and control measures revealed that only WAIS-III-PC total score was significantly correlated with the inappropriate social behavior (r = −0.69; *p* < 0.01) and feelings of embarrassment scores (r = 0.59; *p* < 0.05). 

## 4. Discussions

The primary objective of this study was to compare the performance on our Social Responding Task of individuals with TBI who display ISB with the performance of individuals with TBI who do not display those behaviors. More precisely, this study aimed to obtain preliminary data on our task to verify its usefulness for investigating the neurocognitive processes underlying the inappropriate social behaviors of these individuals with TBI. 

First, we expected TBI-ISB subjects would score significantly higher than the TBI-ASB group and control subjects on a scale measuring the extent to which they would be likely or very likely to display inappropriate social behaviors in a given social situation. This hypothesis was supported by the results. Also, the majority (7/9) of the scenarios associated with an inappropriate behavior were able to contrast the groups. 

Second, we also expected that the TBI-ISB group would score lower than the other groups on the two scales measuring the anticipation of a negative emotional consequence following an inappropriate behavior. This hypothesis was partially supported by the results. Indeed, regarding the results from Part B, no significant difference was found between the groups on the scales measuring the anticipation of a negative emotional consequence following the emission of an inappropriate behavior. However, some scenarios were able to show that significantly fewer TBI-ISB subjects expected feelings of embarrassment after the emission of an inappropriate behavior than did subjects in other groups. These mixed results underline the importance of looking at the specifics of the social situation to understand better the processes associated with the poor behavior choices of the TBI-ISB subjects. 

Finally, the hypothesis of a relationship between the inappropriate behavior scale and the two emotional consequence scales was supported. The results showed that many participants, from each group, who endorsed an inappropriate behavior also failed to anticipate an angry reaction from the other person and/or feelings of personal embarrassment. This was supported by the existence of a negative and significant correlation between behavior and emotional consequence for the TBI-ASB and control groups. However, the results also showed that this relationship was not significant for the TBI-ISB subjects, and that, most of the time, failure to anticipate a negative consequence was not the main reason behind their poor behavior choice. 

Taken together, the results of the present study suggest that a self-reported likely response to hypothetical social scenarios can be a relevant approach to study the neurocognitive processes behind the poor choice of behavior of individuals with TBI-ISB. Also, the results showed that the TBI-ISB participants were likely to endorse an ISB despite being able to anticipate a negative emotional response in themselves or others, suggesting that there were other explanations for their poor behavior. Therefore, in the following section, referring to the literature on mechanisms associated with social decision making, we explore various alternative hypotheses that may help explain the results of our task and guide future studies and instrument development. 

### 4.1. Underlying Mechanisms Affecting Social Behaviors After a TBI

Decision making is defined as choosing from among several alternatives after having considered the consequences associated with each one [34]. The usual tasks for assessing decision making, such as the IGT, present several options from which subjects must choose. In contrast, the Social Responding Task presents subjects with only one option. In fact, after having read the scenario and one behavioral option, subjects are asked to indicate whether they would display the behavior that is presented. It is thus up to the subjects to search their memories for other possible behavioral options in relation to the given situation and to compare those with the option presented, in order to assess the likelihood of their displaying that behavior. It is fair to say that social decision making in everyday life works in a similar way, *i.e.*, faced with a given situation, an individual generally needs to search for behavioral alternatives from within a pool of possible responses [35]. Thus, the first hypothesis would be that the TBI-ISB subjects had no access to other behavioral options, and so found it difficult to make a socially judicious choice. One cognitive mechanism that might explain this lack of access to more socially well-adjusted behavioral alternatives might be a lack of dominant response inhibition. A study by Billieux *et al.* [36] showed that weak inhibition of a dominant motor response, particularly in the presence of emotional stimuli, was linked with a tendency to take poor decisions in gambling tasks. In the case of our task, it may be that, faced with the proposed behavior responding to a particular need, the TBI-ISB subjects found it difficult to inhibit the dominant response, which was to respond to the need, and thereby lost the ability to access and think about other options. However, in the present study, the fact that there was no difference between the TBI-ISB and TBI-ASB groups on the estimation of the presence of perseveration during the task as well as on any of the impulsivity dimensions of the UPPS Impulsive Behavior Scale would argue against a response inhibition disorder. However, regarding impulsivity, a potential lack of introspection among TBI-ISB subjects in the present study might explain the lack of difference between the groups on the dimensions of the UPPS Impulsive Behavior Scale [30]. New studies measuring self-awareness would help to clarify the question. Another potential mechanism that might explain the difficulty in accessing other behavioral options could be a memory retrieval disorder, a frequent cognitive sequela of TBI [37], which would prevent the subject from accessing memories of other potential behaviors in similar social situations. Because the subjects’ mnesic capacities were not measured in the present study, we cannot rule out this potential explanation. However, it has been shown that problems with decision-making in individuals presenting aberrant social behaviors may be dissociated from intact mnesic capacities [1,2]. 

Aberrant social behaviors following frontal lobe injury or resulting from a TBI have been associated with a loss of knowledge of social rules [38], problems judging whether a behavior that might provoke anger in another is socially acceptable [25], or even an inability to detect *faux-pas* type inappropriate social behaviors in a social situation [22]. A second hypothesis to explain the performance of the TBI-ISB subjects in the present study might be that these subjects present a deficit in social judgment that prevents them from recognizing whether a behavior is acceptable or not in a given social situation. A related hypothesis would be that the TBI-ISB subjects had generalized difficulty comprehending the scenarios of the task and were consequently unable to assess the fit between the behavior and the situation presented. One argument in favor of this explanation is that many of the TBI-ISB subjects, unlike those in the other groups, indicated that an appropriate social behavior was likely or very likely to provoke an angry response in the other person. This suggests that the TBI-ISB subjects did not distinguish between appropriate and inappropriate social behaviors because they expected other people to be angry at both. Another related explanation would be that, considering their post-TBI history, people get angry at them a lot for reasons they do not completely understand. The significant correlation between the score on the inappropriate behavior scale and the score on the angry reaction scale for TBI-ASB participants supports this interpretation. However, one argument against poor verbal comprehension skill as the explanation for the performance of the TBI-ISB group is that there was no significant difference in performance between the two TBI groups on the control test for verbal abstraction from the WAIS-III (*i.e*., Similarities).

By manipulating the more or less explicit nature of response–outcome contingencies and the rules governing gains and losses in gambling tasks, it was demonstrated that persons with TBI had difficulty taking decisions in both situations of risk and situations of ambiguity [21]. In situations of risk, the outcomes of decisions can be estimated based on explicit and well-defined probabilities. In the study by Bonatti *et al.* [21], subjects were asked to choose between winning (or losing) a safe, small amount and taking a risk (gambling) for a much larger amount. The chances of winning, in terms of probabilities, were explicitly stated in each trial. Compared to controls, subjects with TBI gambled more frequently in low probability conditions and less frequently in high probability conditions. All in all, subjects with TBI had overall difficulty making advantageous decisions that reflected the probability of winning, and this difficulty was associated with poor performance, particularly, on measures of cognitive assessment and cognitive flexibility. 

A third hypothesis to explain the results of the present study is that the TBI-ISB subjects were inclined to present risky behaviors because they had difficulty assessing the potential risks of an action. In the Social Responding Task of the present study, as in real life, the risks that behaviors would have negative consequences for oneself and for others were more or less explicit and had to be assessed primarily from the social indicators inherent in the situation. Given that the TBI-ASB subjects had results similar to those of the control group, it is conceivable that the subjects without ISB found it easier to assess risks from social indicators than from indicators expressed as probability ratios, as was the case in the gambling task. It may be that the socio-affective nature of the indicators made it possible to call up other mechanisms to support decision making, such as empathic capacities, value attributed to good social relationships, *etc.* However, despite these social indicators, the TBI-ISB subjects still presented risky behaviors. It could be these subjects were less sensitive to these indicators, and that this in itself made it difficult for them to assess the risks associated with ISB. 

Floden *et al.* [39] developed an experimental procedure to separate impulsivity, defined as the tendency to respond immediately to a stimulus, from risky behaviors, defined as the preference for responses associated with low probability of gaining a large reward. These authors showed that risky behaviors, and not impulsivity, were associated with orbitofrontal and left ventrolateral lesions and with reduced behavioral correction following a negative consequence (*i.e*., a loss). According to the somatic markers hypothesis, the ventromedial region, which receives information from several regions of the brain involved in cognitive and emotional processing, is critical for experiencing somatic and emotional responses in complex social situations and for guiding social behavior and optimal decision making [40]. Persons in whom this region is damaged in adult age would have had the chance to acquire knowledge about situational-behavioral response contingencies and to learn moral rules, but would nevertheless present complete insensitivity to the future consequences of their own actions [41]. This insensitivity would be due to emotional hyporeactivity or to difficulty in anticipating/generating an emotional response comparable to the response associated with this situation (or a similar one) in the past. In the present study’s Social Responding Task, it is reasonable to believe that social situations combined with inappropriate social behavior had been, in the subjects’ past, marked by negative values and that the subjects would be able to call up the real or imagined experience of this value (*i.e*., feeling) when reading the scenario. In fact, subjects in the TBI-ASB and control groups indicated they were likely or very likely to experience feelings of embarrassment if they displayed ISB. However, significantly fewer TBI-ISB subjects reported such feelings on at least three of the nine scenarios with ISB. An explanatory mechanism for the difficulty in assessing risks based on social indicators in the TBI-ISB subjects might be difficulty in generating negative feelings that could help inhibit a socially disadvantageous decision. One argument against this potential explanation is that significantly more TBI-ISB subjects also reported feelings of embarrassment in at least two of the three scenarios followed by an appropriate social behavior. This suggests not only that the subjects were able to generate feelings of embarrassment, but that they did so even in circumstances where the behavior in question had probably not been associated with such feelings in the past. We will return later to this inconsistency in behavior–response contingencies.

A prerequisite step in decision making is the clarification of social goals, *i.e.*, the desired outcome for the interaction [42]. Another mechanism that might explain the TBI-ISB subjects’ tendency to choose risky behaviors could be that their decision making is based on establishing non-social goals (*i.e*., serving themselves first) in their social interactions. However, the results on the appropriate behavior scale, which suggested that these subjects would be just as likely or very likely to display the appropriate social behaviors as the other subjects, counter this explanation. Conversely, it might be that the TBI-ASB and control groups’ lower scores on the inappropriate social behaviors scale, as compared with the TBI-ISB group, did not so much reflect their actual decision, but rather their pro-social goal, biased by stronger social desirability. An argument against this explanation is that the two groups of subjects with TBI had similar scores on the Marlowe–Crowne Social Desirability Scale.

The performances obtained on the IGT by subjects with TBI showed they had difficulty in taking advantageous decisions under conditions of uncertainty, *i.e.*, that they selected from the piles of losing cards more often than did the control subjects [20,21]. In situations of ambiguity or uncertainty, information on the probability of winning or losing is missing or contradictory, so the expected utility of the different options cannot be calculated. These difficulties are due to problems with optimal processing of feedback, when it is difficult for executive functions to maintain an advantageous strategy or to flexibly change a strategy that no longer works. In our Social Responding Task, no feedback was given to the subjects. The uncertainty came instead from conflict between satisfying one’s own needs *versus* those of the other. A fourth hypothesis on the results for the TBI-ISB subjects would be that these subjects had difficulty making decisions under conflictual conditions. As such, it may be that their disadvantageous social decision making was due to difficulties in regulating their emotional response when it conflicted with maintaining satisfactory interpersonal relationships. Koenigs and Tranel [11] used a task called the “Ultimatum Game” to study the effect of a ventromedial cortical lesion on decision-making in socially frustrating situations. In this task, two players have the opportunity to share a sum of money. One player offers a portion of that sum to the other. The latter can accept or refuse the offer. If he accepts, the two players share the money as agreed. If he refuses, both players get nothing. It was shown that subjects tended to reject the offer irrationally (since then they received nothing) when the amount offered was small, because this elicited feelings of injustice and anger. The authors of the study [11] demonstrated that the rate of irrational rejection was significantly higher in subjects with ventromedial lesions than in the comparison groups, which suggested to the authors that the ventromedial region is needed when modulation of the emotional response is critical for decision making in a conflictual situation (*i.e*., conflict between financial considerations and the emotional response of frustration). An argument against this explanation is that, in our study, significantly more TBI-ISB subjects indicated they would be likely or very likely to display the inappropriate social behavior in some scenarios where conflict between satisfaction of their own needs and those of others was much less present (e.g., Scenario 4). 

A final hypothesis to explain the results for the TBI-ISB subjects would be a breakdown in the regulation processes attributed to the frontal lobe. Shallice *et al.* [43] posited the existence of two systems of neurological control, *i.e.*, the posterior system, which is involved in direct activation of behavioral routines by the relevant perceptual stimuli, and the supervisory system of the frontal lobes, which monitors and selects the relevant behavioral schemas and inhibits irrelevant ones when new responses are required. According to Cicerone and Tanenbaum [44], the frontal system may be activated by automatic processes similar to somatic markers that signal the triggering of a routine that is inappropriate for the given situation. These authors suggested that breakdown of the frontal regulation system can be seen in social situations that require integrating multiple potential interpretations and associations from all the information available before selecting the appropriate response. Along the same lines, executive functions have been associated with both deficits in decision making [20] and what are called externalizing behaviors (e.g., irritability, impulsivity, insensitivity, inappropriate social behaviors, *etc.*) following TBI [23]. Based on a theory that voluntary regulation of the emotions could be seen as a subset of the frontal attentional system, Rochat *et al.* [23] verified the hypothesis that an executive function deficit might play a role in the development and maintenance of socio-affective disorders observed post-TBI. These authors administered a series of tasks to measure specifically and ecologically the executive functions of subjects with TBI and compared their performances to socio-affective changes observed by persons close to the subjects. The results showed that the Modified Six Elements Test was the only task significantly correlated with the score for changes in externalizing behaviors. According to Burgess [45], the Modified Six Elements Test measures the processes involved in multitasking situations, and among these [23] are several executive function skills required in situations where few exterior constraints are imposed, such as shifting between mental sets or task, updating and monitoring working memory contents, inhibiting prepotent responses, and being able to flexibly allocate attention toward either internal representations (*i.e.*, stimulus-independent affects or thoughts) or external information. In addition, according to Floden *et al.* [39], risky behaviors following a frontal ventral lesion may be due to a general impairment in setting stimulus-response criteria and in the ability to flexibly modify those criteria based on experience. This attentional function of the supervisory system would be important in establishing contingencies [46]. 

Subjects in the TBI-ISB group in the present study may have experienced difficulties on several of these skills in the Social Responding Task. Examples of this include maintaining in prospective memory the intention to return to a topic of conversation begun with friends before being interrupted (Scenario 11), selecting that the relevant information is that my friend wants the same restaurant meal as I do, *versus* the fact that there is only one serving left (Scenario 1), and inhibiting an angry response after being disturbed, in order to grasp in a less emotional way that the person who has just disturbed me (a homeless person) is in need (Scenario 2). Furthermore, these executive deficits could make it difficult to establish appropriate contingencies between behavior and emotional consequences. Indeed, significantly more TBI-ISB subjects indicated they would expect angry responses and/or feelings of personal embarrassment in all three scenarios with appropriate social behaviors. Given the difficulties of integrating the many possible associations between all available pieces of information in a complex social situation, it may be that emotional consequence is not associated with the subject’s behavior, but rather with an exterior social indicator. For example, in Scenario 10, the other’s anger would not be attributed to the behavior as such, which is, in any case, quite appropriate (*i.e*., suggesting to your friend that you get tickets to a different movie), but rather to the fact that only one ticket is left. Embarrassment can be conceived as the feeling that one is in an awkward or shameful position in relation to another’s critical scrutiny or anger [25]. In the Social Responding Task scenarios, feelings of embarrassment are to some extent the corollary of angry responses in others. Consequently, one potential explanation for the results showing that significantly fewer TBI-ISB subjects anticipated feelings of embarrassment for three scenarios with an inappropriate social behavior than did other subjects would be that they are less inclined to experience such feelings because of their difficulty in establishing contingencies between others’ angry responses and their own behaviors. Having attributed the angry response to an indicator outside of their behavior, they would therefore feel no responsibility for that response and would not be embarrassed by their behavior. According to this view, the lack of feelings of embarrassment that could activate the frontal supervisory system and inhibit an ISB would be secondary to a deficit in one of these executive skills. Another argument supporting this explanation is that the TBI-ISB subjects performed significantly less well than those with TBI-ASB on the Picture Completion subtest of the WAIS-III, a test known to be sensitive to problems in regulating visual attention to select essential information and set aside non-essential information. The fact that this WAIS-III subtest was significantly related with inappropriate social behavior, as well as with feelings of embarrassment, is also consistent with this explanation.

### 4.2. Study Limitations

The results of the present study should be interpreted with caution for three main reasons. First, the scenarios of the Social Responding Task incorporate many socio-affective factors that are representative of everyday life but whose influences on decision-making are not yet completely known. Different social circumstances (e.g., conflictual *vs.* non-conflictual, ambiguous *vs.* unambiguous) would need to be studied under carefully controlled conditions in order to understand the precise reasons behind socially disadvantageous decisions. In addition, validation studies are needed to establish the convergence between the Social Responding Task and the inappropriate social behaviors observed in everyday life (e.g., OBS) and decision-making tasks (e.g., IGT). Moreover, the length of the task and fatigue might explain the lack of completion of the task for some subjects. Second, one limitation of the study is the small sample size. Other studies with larger samples are needed to replicate and validate our results with more powerful parametric statistics. Larger samples would also facilitate matching groups in terms of gender and education, which is particularly important when studying emotion and social behavior. Third, given the tenuous participation of the TBI-ISB subjects, there were several control measures that could not be taken, leaving the door open to several alternative explanations for the results obtained. For example, other deficits related to socio-cognitive functions such as empathy or the theory of mind might explain the results from the TBI-ISB subjects. Finally, because it was not possible to gather information from therapists to complete the OBS for the TBI-ASB participants, this group could not definitively be characterized as not having inappropriate social behavior. Thus, it is possible that the “ASB” group is really the “less ISB group.” This concern is moderated by the fact that, if such were the case, it would tend to underestimate rather than exaggerate group differences.

## 5. Conclusions

The present study offers a more systematic approach than descriptive clinical measures for better understanding of the difficulties experienced in social situations by persons with TBI who have inappropriate social behaviors. The results suggest individuals with TBI who present inappropriate social behavior show more impairment in social decision making than do individuals with TBI who present appropriate social behavior. One plausible underlying mechanism affecting social behaviors after a TBI might be a breakdown of the frontal regulation system, which can be seen in social situations that require integrating multiple potential interpretations and associations from all the information available before selecting the appropriate response. We hope the results of the present study will stimulate research and instrument development to explore the still little-known causes of one of the most frequently seen sequelae of TBI, *i.e.*, inappropriate social behaviors. 

## Supplementary material: 12 scenarios from the Social Responding Task

**With the behaviors that were retained for analysis**

***Inappropriate social behavior***

**Scenario 1**

You go out to eat at a restaurant with a friend. When the waitress arrives to take your order, you and your friend decide to order the same daily special. A few minutes later, the waitress returns to tell you that there is only one serving of this daily special left. She apologizes and asks whether you or your friend would like to choose another dish. **Behavior:** I ask my friend to choose something else.

**Scenario 2**

You’re going home after a day at work. During your commute, you’re constantly solicited by homeless persons. After a while, a person who is selling a newspaper aimed at raising people’s awareness of the problem of homelessness asks if you’d like to buy a copy of the newspaper. **Behavior**: I yell at him, telling him I’ve had enough of being disturbed by homeless persons.

**Scenario 3**

A work colleague tells you he has been given two free tickets to a show and invites you to join him. He gives you your ticket and tells you he’ll meet you in the lobby of the theatre. When you tell this news to your girlfriend/boyfriend, you see that she/he is a little disappointed. **Behavior**: I call my work colleague to tell him I was able to exchange the ticket he gave me for two others, and I go to see the show on my own with my girlfriend/boyfriend.

**Scenario 4 **

A friend registers you for a conference on vocational transition and career change. He tells you this conference could be very useful to you in your work. In the lobby of the conference hall, you are given a name tag in your name and a document summarizing each of the presentations. **Behavior**: At the beginning of the afternoon, when there are still two presentations left to attend, I decided to leave and return home.

**Scenario 5**

Friends invite you to a hockey game. But your budget is very tight and you have several payments to make in the coming weeks. You say you’ll think about it and will call them back. **Behavior**: I decide not to go to the hockey game, and I don’t call my friends to tell them about my decision.

**Scenario 6**

You’re invited to a Christmas party at the home of some friends. They ask you to bring some music CDs for the occasion, because they know you have an impressive music collection. However, you particularly enjoy classical music and especially opera. **Behavior:** I arrive at the party with several CDs of opera, which I like very much.

**Scenario 7**

You’re waiting in line at the supermarket to pay for your purchases. There are a lot of customers and only one cash is open. The person behind you tells you she is late in picking up her child at school. A few minutes later, the manager decides to open another cash. You head for the second cash at about the same time as the person behind you. **Behavior:** I take the place ahead of that person.

**Scenario 8**

You decide to go to the movies with your friend to see a police drama. The reviews you read about this movie were good, but they did point out that viewers should pay close attention throughout the movie so as not to lose track of what is happening. **Behavior**: After 30 minutes of viewing, I turn repeatedly to the person sitting beside me to tell him what I think of the movie.

**Scenario 9**

You’ve been standing in the bus for about 15 minutes and you’re tired. A person gets up to get off the bus, leaving an empty seat. You hurry to sit down in that seat. At the same moment, you notice that there is an elderly person with a cane behind you. **Behavior**: I pretend I didn’t see the elderly person and I take the empty seat.

***Appropriate Social Behavior***

**Scenario 10**

A friend suggests going to see a new movie that has just arrived at the cinema. You agree to meet in the cinema lobby. When you get to the ticket counter, the clerk tells you there’s only one ticket left for the movie you’ve selected. **Behavior:** I suggest to my friend that we get tickets to a different movie.

**Scenario 11**

You’re invited to meet new people as part of your school’s alumni reunion. In the evening, while you’re talking with your former classmates, one of them starts passionately recounting the story of an athletic sporting event that you were not involved in. **Behavior**: I listen to my classmate tell the story of the event.

**Scenario 12**

When you’re watching a favourite television program, your neighbour knocks at the door. She explains that she’s having trouble getting to sleep because your television volume is too loud. She asks you politely if you could lower the volume. **Behavior:** I tell my neighbour I didn’t realize the volume was too high, and that I’ll lower it.

*Scale of Part A:*

**Table:**

Likelihood that I would display the behavior			
*0*	*1*	*2*	*3*
Not at all likely	Unlikely	Likely	Very likely

*Scales of Part B:*

**What is the likelihood that: **

**(1) Your friend would be shocked by your behavior?**

**Table:**

*0*	*1*	*2*	*3*
Not at all likely	Unlikely	Likely	Very likely

**(2) You would feel embarrassed by your behavior?**

**Table:**

*0*	*1*	*2*	*3*
Not at all likely	Unlikely	Likely	Very likely
